# Self-Reported History of Childhood Smoking Is Associated with an Increased Risk for Peripheral Arterial Disease Independent of Lifetime Smoking Burden

**DOI:** 10.1371/journal.pone.0088972

**Published:** 2014-02-18

**Authors:** James R. Priest, Kevin T. Nead, Mackenzie R. Wehner, John P. Cooke, Nicholas J. Leeper

**Affiliations:** 1 Division of Pediatric Cardiology, Stanford University School of Medicine, Stanford, California, United States of America; 2 Division of Cardiovascular Medicine, Stanford University School of Medicine, Stanford, California, United States of America; 3 Stanford University School of Medicine, Stanford, California, United States of America; 4 Center for Cardiovascular Regeneration, Methodist DeBakey Heart and Vascular Center, Houston, Texas, United States of America; 5 Division of Vascular Surgery, Stanford University School of Medicine, Stanford, California, United States of America; 6 Cardiovascular Institute, Stanford University School of Medicine, Stanford, California, United States of America; Goethe University, Germany

## Abstract

Atherosclerotic disorders are well known to be associated with obesity, lipid profile, smoking, hypertension and other medical comorbidities, and large cohort studies have explored the childhood correlates to these adult risk factors. However, there has been little investigation into the childhood risk factors for peripheral arterial disease (PAD). We endeavored to better understand the role of smoking in childhood in the risk for PAD in a well described cohort of 1,537 adults at high risk for cardiovascular disease. In a multivariate regression model, we observed an increased risk of PAD among those who reported a history of smoking during childhood (OR = 2.86; 95% CI, 1.99–4.11; P<0.001), which remained statistically significant after controlling for lifetime smoking burden (OR = 1.55; 95% CI, 1.00–2.41; P = 0.049). Our novel observation of disproportionate risk of PAD conferred by a history of childhood smoking may reflect an unrecognized biological mechanism such as a unique susceptibility to vascular injury or an unaccounted for covariate such as secondhand smoke exposure in childhood. This observation suggests further investigation is required into the pathophysiology of smoking in the developing vasculature and the need for detailed clinical data about patterns of childhood smoking and smoke exposure.

## Introduction

While all atherosclerotic disorders share a number of common risk factors, subtle differences are known to exist in the determinants of conditions such as peripheral arterial disease (PAD) and coronary artery disease (CAD) [1]. One such factor is smoking, which remains prevalent in American youth with 23.2% of high school students reporting daily tobacco use in 2011 [2]. To better understand the association of smoking before age 18 and lifetime risk of PAD, we investigated the association of smoking status in childhood with PAD in a well-characterized cohort at high risk for cardiovascular disease [3].

## Materials and Methods

The Genetic Determinants of Peripheral Artery Disease (GenePAD) study comprises individuals (n = 1,755) who underwent an elective, non-emergent coronary angiogram for angina, shortness of breath, or an abnormal stress test at Stanford University and Mount Sinai Medical Centers between January 1, 2004, and March 1, 2008, as previously described [3]–[5]. Participants were included in the study sample if complete data was available on all covariates including age, gender, race, systolic blood pressure, use of anti-hypertensive medication, obesity class, total cholesterol, high-density lipoprotein cholesterol, use of lipid-lowering medication, diabetes, creatinine and CAD (n = 1,537). The Stanford Human Subjects Research Institutional Review Board approved all research described which was conducted under the guidelines of the Declaration of Helsinki, and written informed consent was obtained from all participants.

Before the coronary angiogram, posterior tibial, dorsalis pedis, and brachial artery systolic pressures were measured using a 5-MHz Doppler ultrasound. The ABI for each patient was calculated using traditional methods by dividing the higher ankle pressure of each leg over the higher of the left or right brachial pressures [3]. Each patient was then classified as having PAD by an ABI of <0.9 in either leg or not having PAD with an ABI ≥0.9 in both legs. An experienced cardiologist who was blinded to participant details evaluated coronary angiograms. Hemodynamically significant coronary artery disease (CAD) was defined as >60% stenosis. The use of lipid-lowering and anti-hypertensive medications was evaluated by direct medication inventory. Diabetes status was classified as self-reported use of insulin or oral hypoglycemic agents. Fasting blood (30 ml total) was collected from each patient through a venous or arterial femoral sheath or intravenous line in the arm, processed, then centrifuged at 3000 RPM for 20 minutes at 4°C, and aliquots of EDTA plasma and serum were stored at –75°C. Total and high­density lipoprotein cholesterol were measured by standard assays at the Stanford and Mt Sinai Hospital clinical laboratories, using an AU5400 Chemistry Immuno­Analyzer (Olympus Inc.).

All participants were asked at enrollment, “Have you ever smoked cigarettes daily?”, “At what age did you begin to smoke cigarettes daily?”, “At what age did you stop smoking cigarettes daily?” and “From the time you started smoking to the time you stopped; how many cigarettes, on average, did you usually smoke each day?” The risk of developing PAD was compared in those who reported daily smoking before age 18 (childhood onset smokers), those who started smoking daily after age 18 (adult onset smokers) and never daily smokers. Baseline patient characteristics were compared using a t-test or chi-squared test. Odds ratios (ORs) were calculated using multivariate logistic regression analysis. All models were adjusted for age, gender, race, systolic blood pressure, use of anti-hypertensive medications, and WHO obesity class, total cholesterol, high-density lipoprotein cholesterol, diabetes, creatinine and CAD status. Models were then additionally adjusted for pack-years to test whether childhood or adult onset smoking conferred a risk of PAD independent of lifetime smoking burden. Tests were considered significant if the two-sided P-value was <0.05. All analyses were performed using Stata version 12.0.

## Results

Among the 1,537 participants there were 394 childhood onset smokers, 456 adult onset smokers and 687 never smokers. Compared to adult onset smokers, self-reported childhood onset smokers were younger, more likely to be overweight and obese, consisted of a lower proportion of individuals of Asian descent and had a greater total number of pack years (Table 1). There were 236 (18%) individuals diagnosed with PAD consisting of 96 (24%) self reported childhood smokers, 89 (20%) adult smokers and 91 (13%) never smokers (*P for trend <0.001*).

**Table 1:** Baseline study population characteristics (n = 1,537).

				P-value for difference		
	Self ReportedChildhoodsmokers(n = 394)	Adultsmokers(n = 456)	Neverdailysmoking(n = 687)	P, childhoodvs. adult	P, childhoodvs. never	P, adult vs.never
**Characteristic**						
Age, mean years (SD)	66 (10)	67 (10)	66 (11)	0.050	0.972	0.042
Female, No. (%)	93 (24)	133 (29)	291 (42)	0.067	<0.001	<0.001
Ethnicity, No. (%)						
Caucasian	237 (60)	249 (55)	344 (50)	0.103	0.001	0.133
African	46 (12)	69 (15)	86 (13)	0.142	0.684	0.206
Hispanic	51 (13)	51 (11)	76 (11)	0.431	0.355	0.949
Asian	12 (3)	38 (8)	65 (10)	0.001	<0.001	0.514
Other*	48 (12)	49 (11)	116 (17)	0.511	0.038	0.004
Systolic blood pressure,mean mm Hg (SD)	138 (21)	139 (20)	140 (21)	0.475	0.188	0.555
Use of anti-hypertensivemedication, No. (%)	302 (77)	361 (79)	531 (77)	0.377	0.809	0.454
Obesity class, No. (%)						
Underweight (<18.5 kg/m2)	2 (1)	6 (1)	3 (0)	0.224	0.869	0.100
Normal weight (18.5–24.9 kg/m2)	75 (19)	97 (21)	176 (26)	0.418	0.014	0.091
Overweight (25.0 to 29.9 kg/m2)	150 (38)	211 (46)	279 (41)	0.016	0.411	0.058
Obese I (30.0 to 34.9 kg/m2)	107 (27)	90 (20)	143 (21)	0.011	0.017	0.658
Obese II (35.0 to 39.9 kg/m2)	38 (10)	31 (7)	60 (9)	0.130	0.616	0.237
Obese III (>40.0 kg/m2)	22 (6)	21 (5)	26 (4)	0.516	0.167	0.494
Lipids, mean mg/dL (SD)						
Total cholesterol	140 (37)	136 (37)	139 (36)	0.109	0.867	0.096
HDL cholesterol	39 (12)	40 (12)	41 (12)	0.612	0.007	0.029
Use of cholesterol loweringmedication, No. (%)	250 (64)	306 (67)	460 (67)	0.264	0.243	0.959
Diabetic, No. (%)	118 (30)	144 (32)	204 (30)	0.608	0.930	0.498
Creatinine, mean mg/dL (SD)	1.2 (1.1)	1.2 (1.0)	1.2 (1.2)	0.922	0.963	0.875
Coronary artery disease, No. (%)	316 (80)	373 (82)	483 (70)	0.554	<0.001	<0.001
Cumulative pack-years smoked,mean years (SD)	36 (29)	26 (25)	0 (0)	<0.001	<0.001	<0.001

*Includes Asian-Indian, Pakistani, Middle Eastern and Pacific Islander.

SD; standard deviation; No., number; HDL, high-density lipoprotein; PAD, peripheral arterial disease.

In multivariate regression analyses a significant positive association with PAD was noted for CAD (P<0.001). Consistent with previous observations, a decreased risk of PAD was paradoxically associated with increased obesity class (P = 0.016), while female gender as a covariate was a significant independent predictor of PAD (OR = 2.31; 95% CI 1.68–3.19; P<0.001 [6], [7]. A significant association with PAD was observed (Table 2) for both self reported childhood (OR = 2.86; 95% CI, 1.99–4.11; P<0.001) and adult smoking (OR = 1.67; 95% CI, 1.18–2.37; P = 0.004). Importantly, we continued to observe an association between self reported childhood smoking and PAD even after including total pack-years of smoking in the fully adjusted model (OR = 1.55; 95% CI, 1.00–2.41; P = 0.049). This association was not observed amongst adult smokers (P = 0.782). A direct comparison between self-reported childhood and adult smokers revealed a significant risk difference, after controlling for lifetime smoking burden (P-value for difference = 0.041). Though female gender is a significant independent predictor of PAD there was no statistical evidence that gender is an effect modifier in our study (P>0.473 for interaction terms).

**Table 2:** Risk of peripheral arterial disease by age of daily smoking onset.

		Adjusted*		Adjusted+Lifetime Smoking Burden		
		OR (95% CI)	P-value	OR (95% CI)	P-value	P-value for difference
Never daily smoking		Ref		Ref		
Self Reported Adult smokers		1.67 (1.18–2.37)	0.004	1.06 (0.71–1.58)	0.782	0.041
Self Reported Childhood smokers		2.86 (1.99–4.11)	<0.001	1.55 (1.00–2.41)	0.049	

*Adjusted for age, gender, race, systolic blood pressure, use of anti-hypertensive medication, obesity class, total cholesterol, high-density lipoprotein cholesterol, use of lipid-lowering medication, diabetes, creatinine, coronary artery disease.

OR, odds ratio; CI, confidence interval; Ref, reference.

## Discussion

Smoking is a well-described risk factor for PAD, and here we observe that it may have a particularly deleterious impact on the developing vasculature. The finding that persons who self report smoking during childhood compared to adulthood are at a disproportionately elevated risk for PAD – even after controlling for total lifetime pack-years and the presence of CAD suggests that developing peripheral arteries may be uniquely vulnerable to the endothelial dysfunction associated with tobacco smoke. Alternatively, childhood smoking may potentiate epigenetic changes which later predispose the vessel to PAD. Such long term effects have been observed in the gene expression profile of platelets in ex-smokers, which remain persistently perturbed years later due to smoking-related changes in DNA methylation [8]. Finally, the independent association of PAD and self report of daily smoking before age 18 may also be confounded by unmeasured clinical risk factors, such as exposure to secondhand smoke in childhood, recall bias or one of the emerging, but poorly understood, genetic variants specifically linked with both nicotine addiction and PAD [9].

Our observation of increased PAD among individuals who report daily smoking before 18 years of age, independent of lifetime smoking burden, may reveal unanticipated long-term health and economic benefits of public health interventions designed to reduce tobacco use in the pediatric population and government restrictions on the sale and distribution of tobacco products. This data may also be useful in community practice when counseling adolescents about smoking. Further investigation into the pathophysiology of smoking in the developing vasculature and more detailed clinical data about patterns of childhood smoking and smoke exposure are needed to contextualize this novel observation.
